# Supplementary material for: BNT162b2 induces robust cross-variant SARS-CoV-2 immunity in children
Source: NPJ Vaccines. 2022 Dec 3;7:158. doi: 10.1038/s41541-022-00575-w (PMC9719544; doi:10.1038/s41541-022-00575-w)
Supplement: Supplementary file 1 — Supplemental Figures [file 41541_2022_575_MOESM1_ESM.pdf]

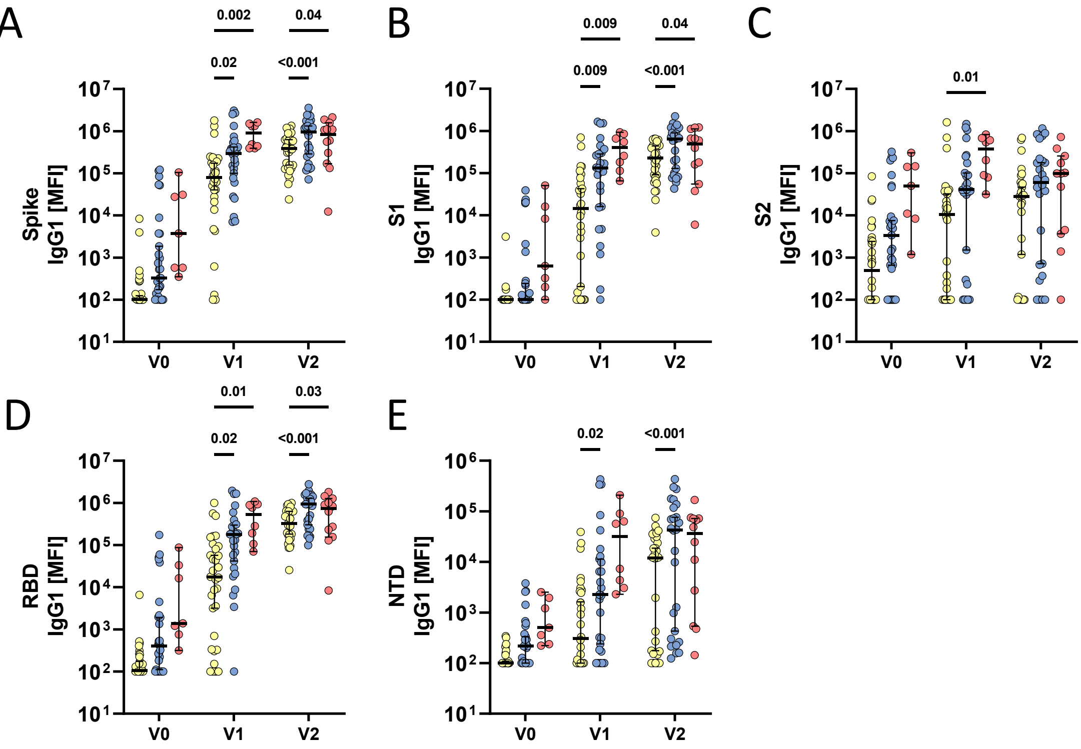

Supplementary Figure 1: Spike domain specific responses after BNT162b2 vaccination. Relative IgG1 binding levels to full length Spike (A, compare Figure 1), S1 domain (B), S2 domain(C), RBD (D, compare figure 1) and N-terminal domain (E, NTD)were determined by Luminex in children receiving either 10 µg of BNT162b2 (ages 5-11 years old, yellow) or 30 µg BNT162b2 (ages 12-16 years old, blue and 16+ years old, red) before ($V0_{10µg\ (5\text{-}11y)}$: 32; $V0_{30µg\ (12\text{-}16y)}$: 29, $V0_{30µg\ (16+y)}$: 7), after the first dose ($V1_{10µg\ (5\text{-}11y)}$: 32; $V1_{30µg\ (12\text{-}16y)}$: 27, $V1_{30µg\ (16+y)}$: 8), or after the second dose ($V2_{10µg\ (5\text{-}11y)}$: 30; $V2_{30µg\ (12\text{-}16y)}$: 26, $V2_{30µg\ (16+y)}$: 11). A two-way ANOVA (two-sided) was used to calculate statistically significant differences between the groups at each timepoint. Exact p-values for statistically significant differences after Benjamini-Hochberg correction for multiple testing are shown above the graph. Horizontal lines indicate the median and error bars the 95% confidence interval.

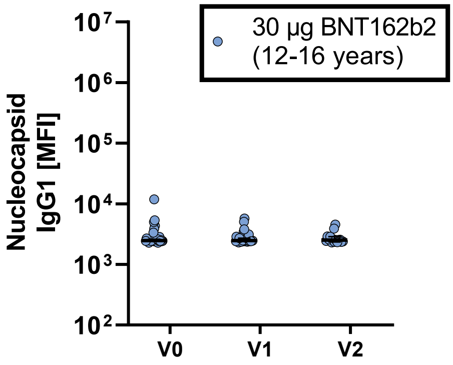

Supplementary Figure 2: Nucleocapsid specific antibody titer show no signs of previous infection. SARS-CoV-2 specific Nucleocapsid IgG1 titers were determined in 12-15 year old individuals before (V0, n=29) and after first (V1, n=29) and second (V2, n=15) dose 30 µg BNT162b2 by Luminex. Horizontal lines indicate the median and error bars the 95% confidence interval.

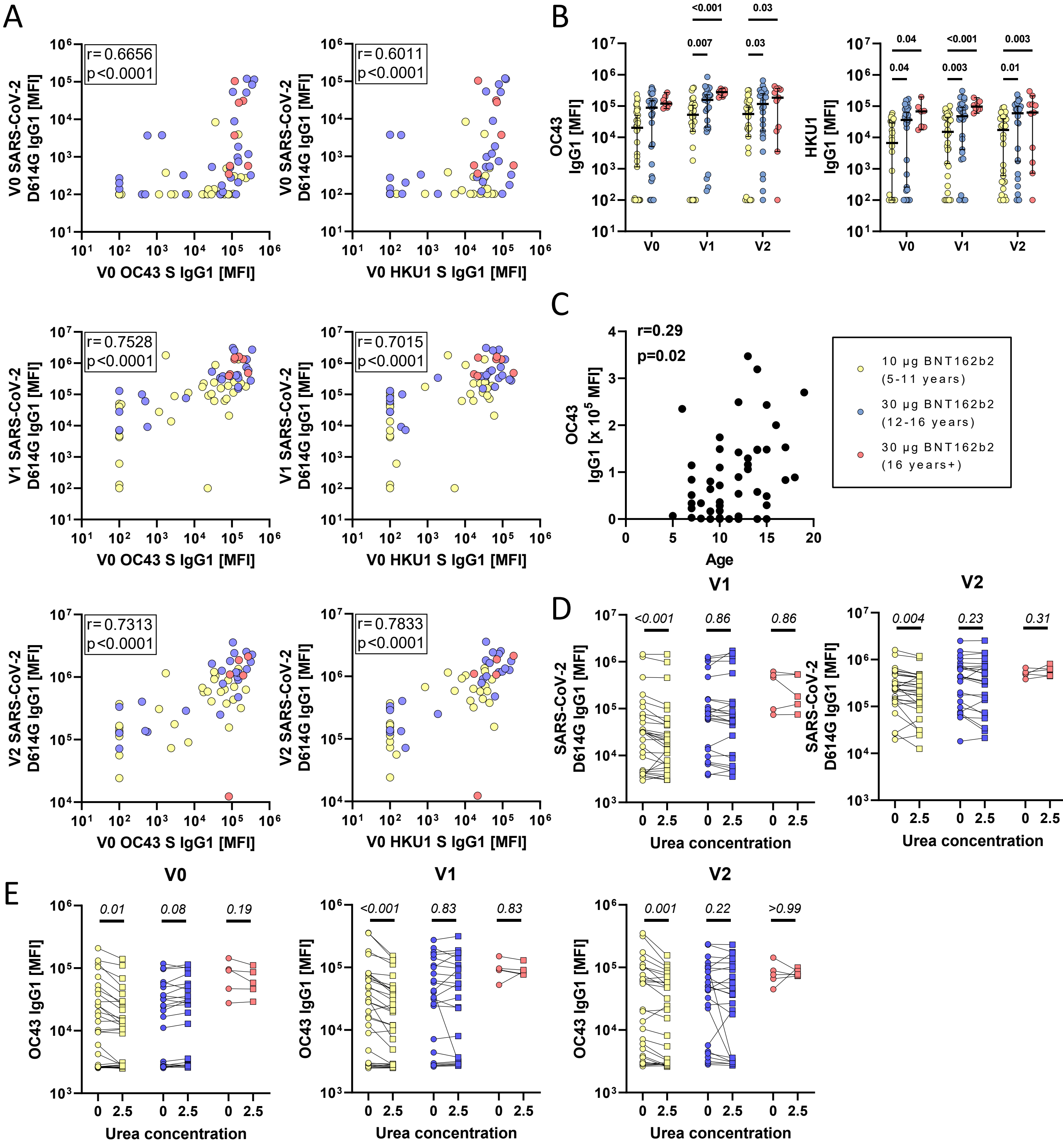

Supplementary Figure 3: Relationship of SARS-CoV-2 and beta-coronavirus titer. A) SARS-CoV-2 Spike specific IgG1 titers at V0 (top), V1 (middle) or V2 (bottom) were plotted against OC43 or HKU1 specific IgG1 titers at V0. Spearman correlations between SAR-CoV-2 Spike and respective beta-coronavirus were calculated and coefficient (r) and p-value indicated in the plot. B) OC43 and HKU1 specific IgG1 titers Luminex in children receiving either 10 µg of BNT162b2 (ages 5-11 years old, yellow) or 30 µg BNT162b2 (ages 12-16 years old, blue and 16+ years old, red) before ($V0_{10µg\ (5\text{-}11y)}$: 32; $V0_{30µg\ (12\text{-}16y)}$: 29, $V0_{30µg\ (16+y)}$: 7), after the first dose ($V1_{10µg\ (5\text{-}11y)}$: 32; $V1_{30µg\ (12\text{-}16y)}$: 27, $V1_{30µg\ (16+y)}$: 8), or after the second dose ($V2_{10µg\ (5\text{-}11y)}$: 30; $V2_{30µg\ (12\text{-}16y)}$: 26, $V2_{30µg\ (16+y)}$: 11). C) Correlation of OC43 specific titers with age. Spearman correlation was calculated and coefficient (r) and p-value indicated in the plot. D-E) Avidity of SARS-CoV-2 (D) or OC43 (E) antibodies was assessed by washing Luminex beads after sample incubation with 2.5 M urea and compared to the native (0M urea) value ($V0_{10µg\ (5\text{-}11y)}$: 30; $V0_{30µg\ (12\text{-}16y)}$: 23, $V0_{30µg\ (16+y)}$: 5, $V1_{10µg\ (5\text{-}11y)}$: 30; $V1_{30µg\ (12\text{-}16y)}$: 24, $V1_{30µg\ (16+y)}$: 5, $V2_{10µg\ (5\text{-}11y)}$: 28; $V2_{30µg\ (12\text{-}16y)}$: 24, $V2_{30µg\ (16+y)}$: 5). Horizontal lines indicate the median and error bars the 95% confidence interval.

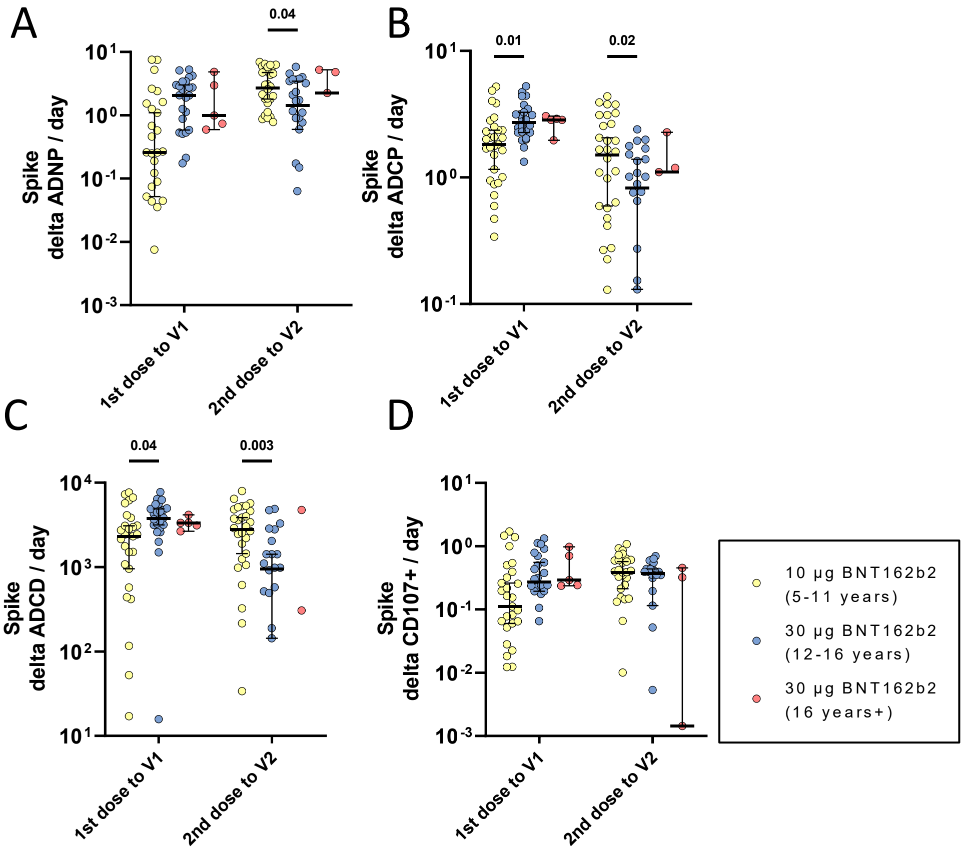

Supplementary Figure 4: Delayed functional responses in children receiving the 10 µg dose. To calculate the daily rate change of distinct Fc effector functions: ADNP (A), ADCP (B), ADCD (C) or ADNKA/CD107a expression of NK cells (D), the difference between the first dose to the next blood draw (at V1) or the second dose and the following blood draw (V2) by the time difference between dose and blood draw was divided, respectively. A two-way ANOVA (two-sided) was used to calculate statistically significant differences between the groups at each timepoint. Exact p-values for statistically significant differences after Benjamini-Hochberg correction for multiple testing are shown above the graph. Horizontal lines indicate the median and error bars the 95% confidence interval.

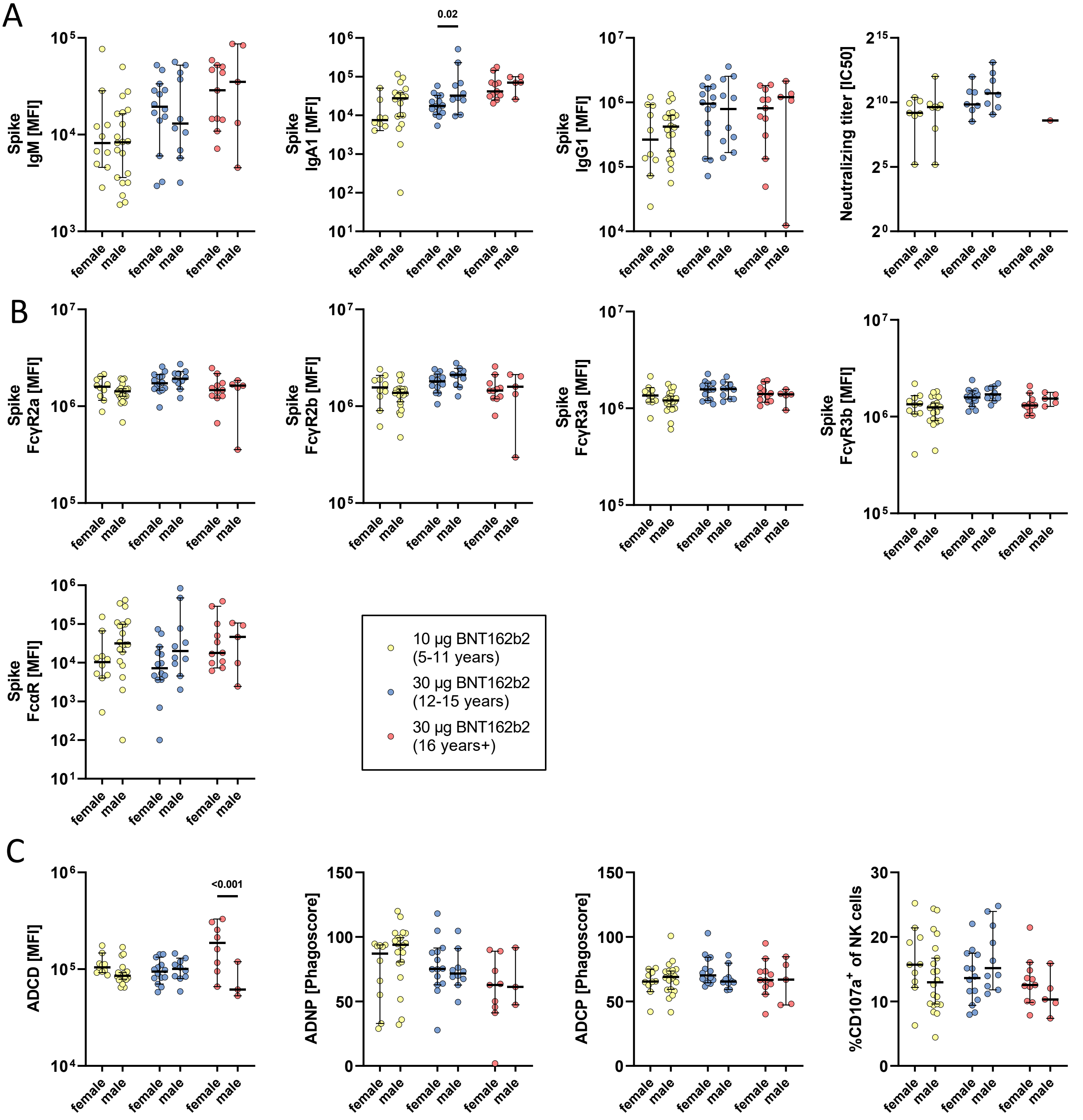

Supplementary Figure 5: Vaccine responses were stratified by sex. Relative SARS-CoV-2 wild-type spike (Wuhan) binding and neutralizing titer (A), Fc receptor binding (B) and Fc mediated functions after two doses of BNT162b2 were stratified by sex (10 µg BNT162b2 in 5-11 years old: n_{female}=10, n_{male}=20, 30 µg BNT162b2 in 12-15 years old: n_{female}=14, n_{male}=10, 30 µg BNT162b2 in 16+ years old: n_{female}=11, n_{male}=5). A two-way ANOVA was used to calculate for statistically significant differences between the sexes. P-values after Bejamini-Hochberg correction for multiple testing of significant comparisons (p<0.05) are indicated. This data is also shown in Figure 1-3. Horizontal lines indicate the median and error bars the 95% confidence interval.

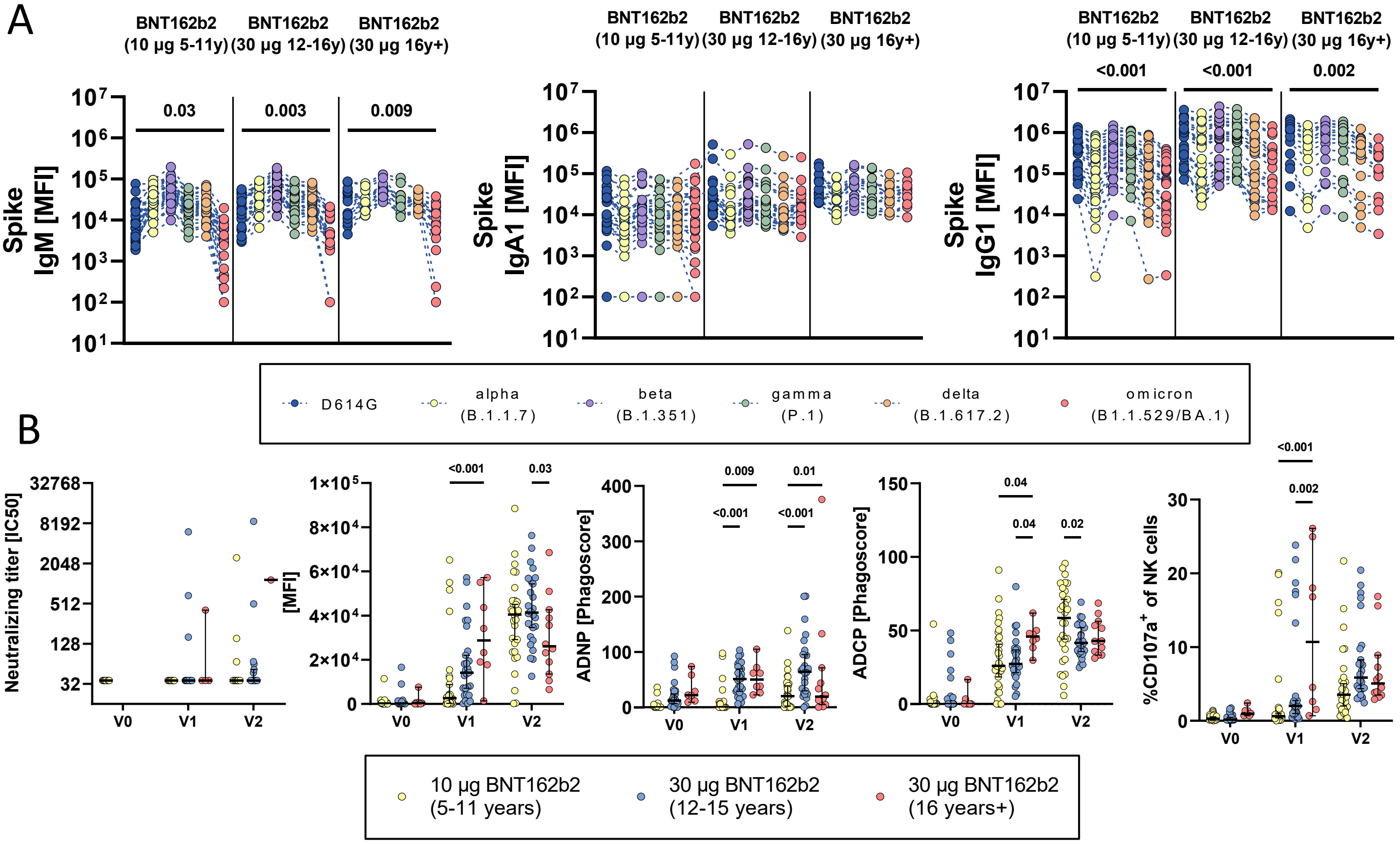

Supplementary Figure 6: Individual VOC specific responses. A) Vaccine induced IgM, IgA1, and IgG1 response to D614G (wild-type; blue), alpha (B.1.1.7, yellow), beta (B.1.1.7, purple), gamma (P.1, green), delta (B.1.617.2, orange), and omicron (B1.1.529/BA.1, red) to the full Spike in children receiving 10µg of BNT162b2 (ages 5-11 years old, n = 30) or adolescent receiving 30 µg BNT162b2 (ages 12-15 years old, n=26) or adults (16+ years old, n=17) at V2. The figure shows individual level data (compare Figure 4A). A two-sided Kruskal-Wallis test with Benjamini-Hochberg correction for multiple testing was performed to compare D614G and omicron specific antibody titers. P-values for significant different comparisons are shown above the dataset. B) The ability BNT162b2-specific antibodies to induce neutralization, complement deposition (ADCD), neutrophil phagocytosis (ADNP), monocyte phagocytosis (ADCP) and NK cell activation by the frequency degranulated CD107+ NK cells against the omicron Spike. A two-way ANOVA (two-sided) was used to calculate statistically significant differences between the groups at each timepoint. Exact p-values for statistically significant differences after Benjamini-Hochberg correction for multiple testing are shown above the graph). Horizontal lines indicate the median and error bars the 95% confidence interval.
