# Supplementary material for: BNT162b2 induces robust cross-variant SARS-CoV-2 immunity in children
Source: NPJ Vaccines. 2022 Dec 3;7:158. doi: 10.1038/s41541-022-00575-w (PMC9719544; doi:10.1038/s41541-022-00575-w)
Supplement: Supplementary file 2 — REPORTING SUMMARY [file 41541_2022_575_MOESM2_ESM.pdf]

nature portfolio

Corresponding author(s): Galit Alter, Lael Yonker

Last updated by author(s): Oct 20, 2022

Reporting Summary

Nature Portfolio wishes to improve the reproducibility of the work that we publish. This form provides structure for consistency and transparency in reporting. For further information on Nature Portfolio policies, see our Editorial Policies and the Editorial Policy Checklist.

Statistics

For all statistical analyses, confirm that the following items are present in the figure legend, table legend, main text, or Methods section.

n/a	Confirmed	
☐	☒	The exact sample size (*n*) for each experimental group/condition, given as a discrete number and unit of measurement
☒	☐	A statement on whether measurements were taken from distinct samples or whether the same sample was measured repeatedly
☐	☒	The statistical test(s) used AND whether they are one- or two-sided<br>*Only common tests should be described solely by name; describe more complex techniques in the Methods section.*
☐	☒	A description of all covariates tested
☐	☒	A description of any assumptions or corrections, such as tests of normality and adjustment for multiple comparisons
☐	☒	A full description of the statistical parameters including central tendency (e.g. means) or other basic estimates (e.g. regression coefficient) AND variation (e.g. standard deviation) or associated estimates of uncertainty (e.g. confidence intervals)
☐	☒	For null hypothesis testing, the test statistic (e.g. *F*, *t*, *r*) with confidence intervals, effect sizes, degrees of freedom and *P* value noted<br>*Give P values as exact values whenever suitable.*
☒	☐	For Bayesian analysis, information on the choice of priors and Markov chain Monte Carlo settings
☒	☐	For hierarchical and complex designs, identification of the appropriate level for tests and full reporting of outcomes
☒	☐	Estimates of effect sizes (e.g. Cohen's *d*, Pearson's *r*), indicating how they were calculated

Our web collection on statistics for biologists contains articles on many of the points above.

Software and code

Policy information about availability of computer code

Data collection	ForeCyt® Standard Edition 8.1 was used to collect Luminex, ADNP, ADCP and ADCD assay.
Data analysis	Microsoft Excel 365 was used to compile experimental data and patient information.<br>GraphPad Prism 9 was used to generate univariate plots and perform statistical analysis.<br>RStudio (v.1.3 and R v.4.0) and the integrated functions in 'systemRology' package (https://github.com/LoosC/systemsseRology) (1.0) were used to perform feature selection and partial least-squares discriminant analysis(PLS-DA) implemented in 'ropls' package (1.22.0).

For manuscripts utilizing custom algorithms or software that are central to the research but not yet described in published literature, software must be made available to editors and reviewers. We strongly encourage code deposition in a community repository (e.g. GitHub). See the Nature Portfolio guidelines for submitting code & software for further information.

Data

Policy information about availability of data

All manuscripts must include a data availability statement. This statement should provide the following information, where applicable:

- Accession codes, unique identifiers, or web links for publicly available datasets
- A description of any restrictions on data availability
- For clinical datasets or third party data, please ensure that the statement adheres to our policy

All relevant data are included in this manuscript. No data was stored externally.

Human research participants

Policy information about studies involving human research participants and Sex and Gender in Research.

Reporting on sex and gender	Sex-based analysis has been included in Supplemental Figure 5.
Population characteristics	- children 5-11 years old (n= 32, median age 9 years, 34 % female) receiving the age recommended 10 µg dose<br>- adolescent 12-15 years old (n= 31, median age 13 years, 65 % female) receiving the age recommended 30 µg dose<br>- adults (n = 20, median age 23 years, 60 % female) receiving the age recommended 30 µg dose
Recruitment	Participants were invited to participate in this study and donate blood samples prior to the first regular vaccination.
Ethics oversight	All study procedures were approved by the MassGeneralBrigham Institutional Review Board (#2020P000955 or #2021P002628).

Note that full information on the approval of the study protocol must also be provided in the manuscript.

Field-specific reporting

Please select the one below that is the best fit for your research. If you are not sure, read the appropriate sections before making your selection.

☒ Life sciences ☐ Behavioural & social sciences ☐ Ecological, evolutionary & environmental sciences

For a reference copy of the document with all sections, see nature.com/documents/nr-reporting-summary-flat.pdf

Life sciences study design

All studies must disclose on these points even when the disclosure is negative.

Sample size	No sample size calculation was performed. We included all samples from pediatric patients who received BNT162b2 vaccine and consented to participate into this study until begin of the analysis in January 2022.
Data exclusions	No data was excluded.
Replication	All experiments were run in replicates.
Randomization	Samples were randomly distributed in 96 well plates
Blinding	Investigators were blinded during data collection. Group allocation had to be revealed to perform the data analysis.

Reporting for specific materials, systems and methods

We require information from authors about some types of materials, experimental systems and methods used in many studies. Here, indicate whether each material, system or method listed is relevant to your study. If you are not sure if a list item applies to your research, read the appropriate section before selecting a response.

Materials & experimental systems

n/a	Involved in the study	
☐	☒	Antibodies
☐	☒	Eukaryotic cell lines
☒	☐	Palaeontology and archaeology
☒	☐	Animals and other organisms
☒	☐	Clinical data
☒	☐	Dual use research of concern

Methods

n/a	Involved in the study	
☒	☐	ChIP-seq
☒	☐	Flow cytometry
☒	☐	MRI-based neuroimaging

Antibodies

Antibodies used	1 .Mouse Anti-Human IgG1-PE (Southern-Biotech, #9054-09, clone:HP6001)<br>2. Mouse Anti-Human IgG2-PE (Southern-Biotech, #9060-09, clone:31-7-4)<br>3. Mouse Anti-Human IgG3-PE (Southern-Biotech, #9210-09, clone:HP6050)<br>4. Mouse Anti-Human IgG4-PE (Southern-Biotech, #9200-09, clone:HP6025)

5. Mouse Anti-Human IgM-PE (Southern-Biotech, #9020-09, clone:SA-DA4)
6. Mouse Anti-Human IgA1-PE (Southern-Biotech, #9130-09, clone: B3506B4)
7. Mouse Anti-Human IgA2-PE (Southern-Biotech, #9140-09, clone: A9604D2)
8. Anti-guinea pig complement C3 goat IgG fraction (MP Biomedical, #855385. polyclonal)
9. anti-human CD66b Pacific Blue (Biolegend, #305112 ,clone G10F5)
10. anti-human CD107a (BD, #328634, clone: H4A3)
11. anti-human CD56 (BD, #335791, clone: NCAM16.2)
12. anti-human CD3 (BD, #300426, clone: UCHT1)
13. anti-human MIP1β (BD, #562900, clone: D21-1351)
14. anti-human IFNγ (BD, #506507, clone: B27)

Validation

All antibodies are well established and quality controlled by the manufacturer. Additional information and references can be obtained on the company websites.
The use of antibodies 1-4 was previously validated: Brown EP, Licht AF, Dugast AS, Choi I, Bailey-Kellogg C, Alter G, et al. High-throughput, multiplexed IgG subclassing of antigen-specific antibodies from clinical samples. J Immunol Methods. 2012;386(1-2):117-23.
Antibody8 was described here: Fischinger, S., J. K. Fallon, A. R. Michell, T. Broge, T. J. Suscovich, H. Streeck, and G. Alter. 2019. 'A high-throughput, bead-based, antigen-specific assay to assess the ability of antibodies to induce complement activation', J Immunol Methods, 473: 112630.
Antibody 9: Karsten, C. B., N. Mehta, S. A. Shin, T. J. Diefenbach, M. D. Slein, W. Karpinski, E. B. Irvine, T. Broge, T. J. Suscovich, and G. Alter. 2019. 'A versatile high-throughput assay to characterize antibody-mediated neutrophil phagocytosis', J Immunol Methods, 471: 46-56.

Eukaryotic cell lines

Policy information about cell lines and Sex and Gender in Research

Cell line source(s)	HEK293T (ATCC)
Authentication	The cell line were not further authenticated.
Mycoplasma contamination	Cell lines were monitored for mycoplasma contamination at ATCC
Commonly misidentified lines (See ICLAC register)	No commonly misidentified cell lines were used.
